# Polyvinyl Alcohol-Based 3D Printed Tablets: Novel Insight into the Influence of Polymer Particle Size on Filament Preparation and Drug Release Performance

**DOI:** 10.3390/ph14050418

**Published:** 2021-05-01

**Authors:** Andrea Gabriela Crișan, Alina Porfire, Rita Ambrus, Gábor Katona, Lucia Maria Rus, Alin Sebastian Porav, Kinga Ilyés, Ioan Tomuță

**Affiliations:** 1 Department of Pharmaceutical Technology and Biopharmacy, Faculty of Pharmacy, “Iuliu Hațieganu” University of Medicine and Pharmacy, 41 Victor Babeș Street, 400012 Cluj-Napoca, Romania; crisan.andrea@umfcluj.ro (A.G.C.); ilyes.kinga@umfcluj.ro (K.I.); tomutaioan@umfcluj.ro (I.T.); 2 Institute of Pharmaceutical Technology and Regulatory Affairs, University of Szeged, Interdisciplinary Excellence Centre, 6 Eötvös Street, H-6720 Szeged, Hungary; arita@pharm.u-szeged.hu (R.A.); katona@pharm.u-szeged.hu (G.K.); 3 Department of Pharmaceutical Analysis, Faculty of Pharmacy, “Iuliu Hațieganu” University of Medicine and Pharmacy, 6 Louis Pasteur Street, 400349 Cluj-Napoca, Romania; lucia.rus@umfcluj.ro; 4 National Institute for Research and Development of Isotopic and Molecular Technologies, 67-103 Donath Street, 400293 Cluj-Napoca, Romania; poravsebastian@gmail.com

**Keywords:** 3D printing, fused deposition modeling, hot melt extrusion, polyvinyl alcohol, residence time

## Abstract

Three-dimensional printing (3DP) by fused deposition modeling (FDM) has gained momentum as a promising pharmaceutical manufacturing method due to encouraging forward-looking perspectives in personalized medicine preparation. The current challenges the technology has for applicability in the fabrication of solid dosage forms include the limited range of suitable pharmaceutical grade thermoplastic materials. Hence, it is important to investigate the implications of variable properties of the polymeric carrier on the preparation steps and the final output, as versatile products could be obtained by using the same material. In this study, we highlighted the influence of polyvinyl alcohol (PVA) particle size on the residence time of the mixtures in the extruder during the drug-loaded filament preparation step and the consequent impact on drug release from the 3D printed dosage form. We enhanced filament printability by exploiting the plasticizing potential of the active pharmaceutical ingredient (API) and we explored a channeled tablet model as a design strategy for dissolution facilitating purposes. Our findings disclosed a new perspective regarding material considerations for the preparation of PVA-based solid dosage forms by coupling hot melt extrusion (HME) and FDM-3DP.

## 1. Introduction

Novel manufacturing technologies, with the adequacy to address the pharmaceutical supply chain disruptions associated with health emergencies such as pandemic events, are vital in order to ensure stable access to treatments. The crisis generated by the COVID-19 outbreak highlighted the requirement for innovative fabrication methods of dosage forms that could ensure rapid response to early indicators, would be readily adaptable to local needs and could be deployable to mobile pharmacies. A uniquely positioned manufacturing method to function as the best fit for ensuring supply chain security and an effective response in healthcare emergency circumstances is three-dimensional printing (3DP), a ground-breaking technology with encouraging potential for pharmaceutical applications. The interest regarding its relevance in medication production was triggered by the Food and Drug Administration’s approval of the first 3D printed dosage form marketed under trademark of Spritam^®^ in 2015 [1]. The five major classes of 3DP technologies are vat polymerization, material jetting, powder bed fusion, material extrusion, and direct energy deposition [2]. Each class includes a number of techniques among which stereolithography, selective laser sintering, fused deposition modeling (FDM), semi-solid extrusion, or binder jet printing have been investigated for applicability in the development and manufacturing of pharmaceutical formulations [3]. However, FDM, a methodology that belongs to the class of material extrusion, proved to be the focus of attention on account of advantages, such as flexibility, simplicity of the process, economic rationality, possibility of modulating drug release through careful selection of materials and processing conditions, etc. [4,5].

Manufacturing by FDM-3DP adapted for the pharmaceutical sector relies on active pharmaceutical ingredient (API) containing feedstock filaments that are melted and extruded throughout a heated nozzle, followed by layer-by-layer deposition of the material on a build plate, according to the preset digital model, until the final object is fabricated [6]. The initial attempts of adjusting the technology depended on commercially available filaments in which the drug was included through immersion into a solution of the API [7,8,9]. The method, however, presented drawbacks, such as achievement of low drug loading, requirement of supplementary processing steps, or reduced reproducibility, and the focus was relocated towards preparation of the drug containing filaments by Hot Melt Extrusion (HME). Throughout the HME operation, the materials are blended and heated in the extruder, the melted mass being subsequently pressed through a die in furtherance of filament shaping, through rapid solidification by cooling. The primary advantage of the methodology is achievement of high drug loading, as preparation of 3D printable filaments with a 50% w/w drug content was reported [10]. In addition, HME provides the opportunity to surpass the solubility issues of poorly soluble APIs through solid dispersion preparation, a system wherein the drug is molecularly disseminated into the carrier matrix [11]. However, according to recently published investigations, similar advantages could be obtained by employing a newly reported printing technique, namely direct powder extrusion 3D printing, which is a single-phase preparation method that eliminates the HME phase [12,13,14].

**Figure 1:**
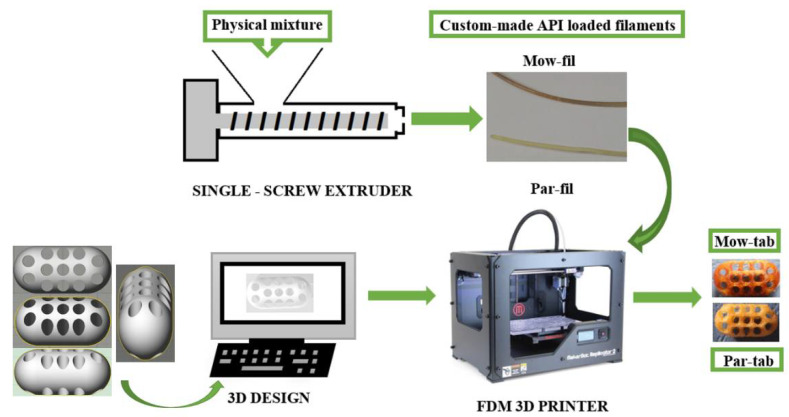
Schematic representation of KET-loaded channeled tablet preparation by coupling HME-FDM-3DP.

The appropriate polymer selection step is critical considering that the matrix forming component represents a significant proportion of the formulation and plays an important role in the feasibility of the Hot Melt Extrusion–Fused Deposition Modeling–Three-Dimensional Printing (HME-FDM-3DP) methodology presented in Figure 1 and in obtaining a favorable printing outcome. Moreover, careful consideration is required if the desired output of the process is an amorphous solid dispersion (ASD), since the polymer’s the capability to stabilize the amorphous API within the system, the miscibility of constituents, the hygroscopicity or readiness for intermolecular interactions have an impact on the production, dissolution performance, and stability of the dosage form [15,16,17].

Polyvinyl alcohol (PVA) is a hydrophilic semi-crystalline synthetic material presenting notable thermoplasticity, an imperative prerequisite for FDM-3D printability. It is biocompatible, water soluble and exhibits ranging characteristics such as melting temperature (T_m_) or viscosity depending on the hydrolysis grade, as the polymer is obtained through hydrolysis of polyvinyl acetate [18,19,20]. However, it was reported that neat PVA is difficult to process through HME due to high processing torque and necessity of elevated temperatures, the resulting filaments being extremely brittle, improper for FDM-3DP [21]. A widely reported method to enhance PVA processability and functionality for HME-FDM-3DP is the addition of suitable plasticizers such as mannitol [22,23] or sorbitol [18,21]. However, the processing aids must be cautiously selected if an ASD is the target formulation, as they may influence the stability of the system by facilitating crystallization and phase separation [24,25]. Therefore, since small-molecule APIs can serve as plasticizers when mixed with polymers, it is appropriate to investigate if the addition of supplementary processing aids into PVA-based systems is avoidable.

PVA is generally utilized as a safe excipient in various pharmaceutical preparations, thus there are diverse available marketed types of the polymer with different properties. A systematic study conducted by Saviano et al. [26] examined the influence of PVA polymer particle size on drug loading and process efficiency for the preparation of 3D printed tablets, demonstrating that finer batches exhibited better processability. However, the extrusion of the smallest particle size stack (<250 µm) could not be performed in this study due to insufficient torque force, and the lately developed PVA product for HME-FDM-3DP applicability (Parteck^®^ MXP) belongs to this category. Consequently, further evaluations are required to investigate other possible implications of PVA particle size on the quality attributes of dosage forms prepared by HME-FDM-3DP.

Previous works addressing the evaluation of PVA based 3D printed tablets revealed slow dissolution rates of the drugs from the dosage forms. For instance, Skowyra et al. achieved 80% release of prednisolone after 12 h [9], while Goyanes et al. [8] obtained 70% release of fluorescein from the 90% infill tablet after 6 h. More recent research evidenced the suitability of PVA for 3DP of products with more rapid drug release [27] and highlighted the impact of infill settings on the dissolution rates of the drug. Thus, higher porosity provided by lower infill (60%) ensured almost complete drug release in 45 min, whereas 90–120 min were necessary for complete API release from dosage forms with 100% infill [18]. Palekar et al. evidenced the major impact of the dosage form dimensions on the release rates [21], as the increase in minicaplet size significantly expanded the required time for the dissolution of the equivalent quantity of drug. Therefore, the challenge of achieving fast drug release from a PVA based drug delivery system with larger dimensions imposed by the necessity to include a high dose of the API must be addressed.

In this work, we report the successful preparation of high drug loaded (30%, *w/w*) PVA-based hot melt extruded filaments suitable for FDM-3DP applications. A novel formulation strategy was investigated by exploiting the plasticizing effect of the API and we demonstrated that the addition of supplementary processing aids that could affect the stability of the products is avoidable. The influence of polymer particle size on the filament preparation phase was assessed, but also the further effects on drug release from the 3D printed dosage forms were highlighted, and new considerations regarding material selection rationale were disclosed. A channeled tablet model was also explored as a design strategy targeted towards enhancing the drug release rates from a PVA-based 3D printed system, impaired by the considerable dimensions, as a substantial effect of the tablet size on the dissolution process was evidenced by previous studies. The model drug employed in this study is ketoprofen (KET), a highly potent nonsteroidal anti-inflammatory drug which is widely prescribed due to its analgesic, antipyretic and anti-inflammatory properties in the treatment of numerous acute and chronic conditions, but mostly in the management of pain and rheumatic disorders. In conformity with the Biopharmaceutics Classification System (BCS), KET is assigned to Class II by presenting poor water solubility and high permeability [28,29].

## 2. Results and Discussion

### 2.1. Material Characterization

PVA is selected as a hydrophilic matrix forming polymer for the preparation of drug loaded filaments, since it is a benchmark water-soluble material for FDM-3DP applications with excellent biocompatibility. Due to the fact that raw material variations represent a significant matter in pharmaceutical manufacturing as they can potentially have an impact on processability and consequently influence the characteristics of the final products, investigation of the same excipient provided by different suppliers is of scientific and practical importance. Therefore, two products provided by different manufacturers but based on the same type of partially hydrolyzed (88%) PVA were evaluated in this work. Thus, the only difference between the selected excipients, Mowiol^®^ 4–88 (MOW) and Parteck^®^ MXP (PAR), is the polymer particle size. PAR is a product specifically designed for applications in HME.

Following initial grinding of MOW, the PVA sort with large particle dimensions, the determination of particle size revealed an arithmetic mean diameter of 612 µm for MOW with an average deviation of 298 µm and 81 µm for PAR with average deviation of 50.8 µm. Through the preliminary studies, two formulations were considered for HME processing. The first contained 30% KET and 70% MOW, while the second one consisted of 30% KET and 70% PAR. Trial HME operations were carried out using the single-screw extruder. The PAR based physical mixture exhibited poor flowability and processability by HME, therefore Aerosil^®^ was introduced in the formulation as a flow improvement additive. The addition of Aerosil in the MOW based formulation was not necessary since it exhibited proper flowability for the feasibility of the extrusion phase. Consequently, the blends (Mow-mix and Par-mix) with the qualitative and quantitative formulas presented in Table 1 were selected for further investigations.

It was previously reported that the PVA polymer particle dimensions have a significant influence on the homogeneity of the mixture with the API and on the extrusion procedure using a single screw assembly, finer batches presenting improved processability [26]. However, the extrusion of the smallest particle size batch (<250 µm) was not performed in the referenced study. Additionally, it is well known that the powder flow properties are reliant on the particle size of the materials [30]. The flow characteristics of MOW, PAR, Mow-mix and Par-mix were investigated by assessing the bulk and tapped densities, followed by calculation of Carr’s index and Hausner ratio. The results presented in Table 2 revealed excellent flowability for MOW, as the Carr’s index and Hausner ratio were found to be 8% and 1.09, respectively, while the values registered for PAR were 20% and 1.25, which correspond to fair flowability. Determinations performed on the physical mixtures revealed that the flowability of the blends was negatively affected by the addition of KET, as Mow-mix presented fair flow properties, while Par-mix exhibited passable flowability in spite of the fact that Aerosil^®^ was included as a glidant into the formulation. Ensuring proper flowability of the physical mixtures intended for processing by HME is essential, particularly when these consist of API included in high proportions and a polymer with small particle size, both with detrimental influence on the flow properties of the blends. These combinations are not ideal for feeding the extruder, as our attempts of processing KET-PAR blends prior to the inclusion of Aerosil as a glidant revealed.

Previous studies conducted using PAR did not highlight issues concerning flowability of the material during HME, presumably due to employment of twin screw extruder configurations [18,21,27] with advantages over single screw constructions such as positive conveying, superior mixing, and inhibition of substance adherence to the screws [11,31].

### 2.2. HME of KET-Loaded Filaments

Schematic representation of the overall HME-FDM-3DP workflow is displayed in Figure 1. The extrusion process of the two formulations, Mow-mix and Par-mix, respectively, was carried out at 180 °C, a parameter that was established following processability assessment at various temperatures. High drug loaded (30% *w/w*) filaments were prepared. The possibility of including an increased amount of the API in the filament by extrusion is one of the main advantages of the technique. Numerous studies explored the alternative API-loading technique of commercially available PVA filaments by the passive diffusion approach [7,8,9]. However, the most important limitation of the method is the achievement of very reduced drug loading. In a recently published work, Cerda et al., reported a 2.2% drug loading of PVA filaments by passive diffusion [32]. In the referenced study, tablets with an average weight of 670 mg were printed (100% infill density) in order to include a dose of 15 mg of the API. Therefore, the applicability of passive diffusion methodology for the preparation of API-loaded 3D printable filaments is restricted to extremely potent drugs. Additionally, incomplete drug release (~20%) over 24 h was obtained according to the presented data. Consequently, as it was previously demonstrated that the drug dissolution from PVA-based 3D printed systems is highly dependent on the dimensions and the porosity of the dosage forms [21], the high drug load granted by preparation of filaments by HME enables flexibility in adjusting these features towards obtaining tailored products. Moreover, the concern of the materials used for the preparation of the commercially available PVA filaments must be addressed, as the quality and safety of the final product depends greatly on it. By contrast, HME is applied for the development of high-quality filaments based on pharmaceutical grade excipients. Furthermore, in the specific context of PVA based systems, by selecting the appropriate sort with specific attributes, i.e., particular molecular weight, the release profile of the final 3D printed dosage form could be influenced from the filament preparation step through careful material considerations. Thus, the HME technique preserves its superiority for manufacturing drug containing 3D printable filaments.

**Figure 2:**
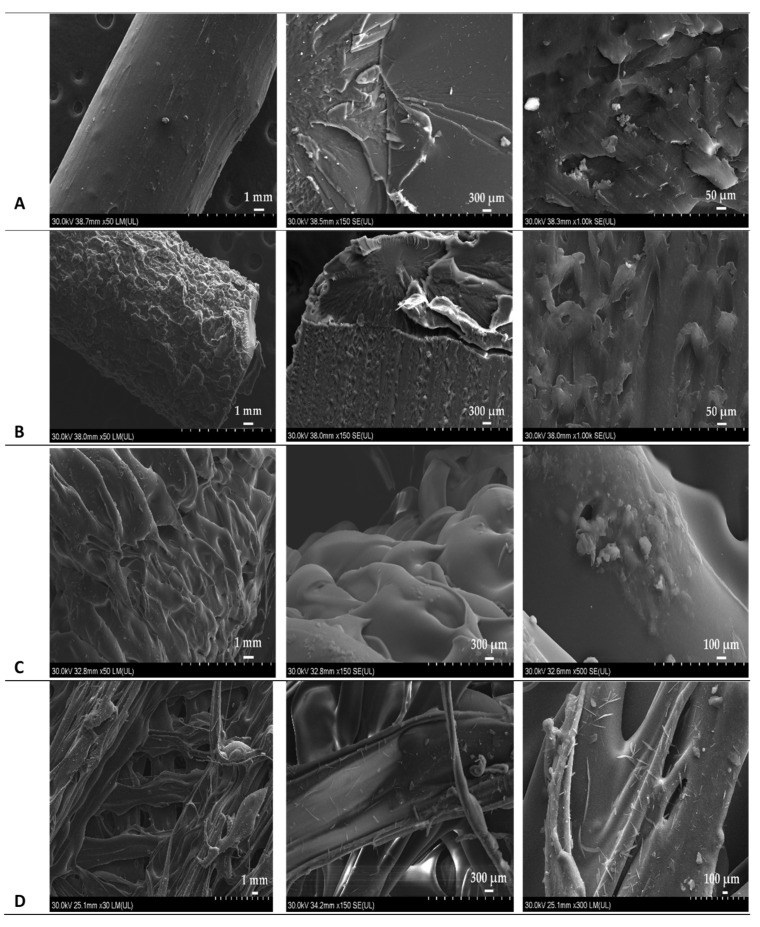
SEM images of drug loaded filaments: (**A**) Mow-fil; (**B**) Par-fil; and 3D printed tablets: (**C**) Mow-tab; (**D**) Par-tab.

An important aspect to be taken into consideration regarding HME is the amount of time spent by the materials in the extruder, also known as residence time, as it has a significant influence on the stability of the substances, the polymorphic transformations and the aspect of the extrudates [31]. Important differences regarding the residence time of the two formulations occurred throughout the extrusion phase, taking into account that the interval of time measured from the moment of feeding the physical mixture into the apparatus to the filament discharge initiation was nearly 8 min, in the instance of Mow-mix, and less than 3 min for PAR-mix. The variation is assignable to the differences in terms of polymer particle size, as the reduced dimension of PAR particles ensured a more rapid melting of PAR-mix and, as a consequence, an accelerated initiation of discharge of the melted mass was achieved. Therefore, it is noticeable in Figure 1 that the obtained filaments, Mow-fil and Par-fil, exhibited aspect dissimilarities, a darker colored product being acquired in case of Mow-fil. Scanning electron microscopy (SEM) investigation of the prepared KET-loaded PVA-based filaments (Figure 2A,B) exposed compact HME products with a certain degree of surface roughness.

It is known that HME of neat PVA generates brittle filaments which are not adequate for 3DP on grounds of inappropriate flexibility and mechanical properties [21]. As discussed earlier, this limitation is surpassed by the addition of plasticizers. In our case, however, the plasticizing function was fulfilled by the API. KET is a small molecule with Tm ~94 °C and its suitability as a plasticizer was previously demonstrated [33,34]. Therefore, the molecular friction within the large, entangled PVA chains was lowered during thermal treatment [4], and the viscosity of the melted mass was reduced by the liquid state of the API due to higher processing temperatures compared to its melting point. Thus, a molecular reorganization was enabled [21], that enhanced the extrusion process and ensured the printability of the filament. The chosen proportion of the drug utilized as a non-traditional plasticizer of PVA has proved to be adequate. Deficiencies such as cavities and aggregations resulting from low API content and subsequent poor flowability of the melted mass, or decrease in solidification speed of the layers due to excessive reduction in the melt viscosity by an overly elevated KET content, that could have negatively impacted the printing process and the quality of the fabricated dosage form [35] were avoided. Highlighting the possibility of exploring the plasticizing potential of the API is one of the aspects of novelty in this study. Avoiding the addition of supplementary processing aids is particularly important when the preparation of an ASD is the target, since plasticizers might have a negative impact on the stability of the system by facilitating crystallization and phase separation [24,25].

### 2.3. 3D Printing of Channeled Tablets

As previously mentioned, the composition of the feedstock filament influences its mechanical properties and ultimately its printability [36]. In our case, both types of filaments, i.e., Mow-fil and Par-fil, were suitable for FDM-3DP by presenting appropriate stiffness, an essential attribute since the filament functions as a piston which forces the melted drug-polymer mass across the nozzle, as well as adequate ductility, in order to resist the bending forces during the feeding process. These presumptions are based on the successful execution of the printing process considering that the mechanical properties of the filaments were not investigated in this study.

The custom-designed channeled tablets (Figure 1), with an architecture approach that is similar to the one previously reported by Sadia et al. [37], were prepared by deposition of the liquefied mass obtained through filament melting. The process parameters were carefully selected to ensure suitable viscosity of the melted mass and deposition of layers lacking width deviations or air bubbles that could alter the accuracy of the API dose in the final product. Although in previous publications it was reported that significantly higher temperatures are required for the printing process compared to the extrusion step due to reduced residence time of the materials in the heated nozzle and limited shear [38,39], in this case a slightly higher temperature (185 °C) was adequate as the API included in high proportions (30% w/w) fulfilled plasticizing functions, lowered the required processing temperature, and improved the flow of the melted mass.

The outlying morphological characteristics of the dosage forms were examined utilizing SEM. Micrographs of the Mow-tab (Figure 2C) did not evidence the presence of crystals on the surface. In contrast, examination of the Par-tab (Figure 2D) revealed small crystals on account of the presence of Aerosil.

Since drug release rates from polymeric matrices are slow and decelerate by increasing the volume of the dosage form [37,40], integrated channels with a diameter of 2.3 mm were exploited as a design strategy to enhance drug release from the polymer-abundant structure by amplifying the surface area/volume ratio. The approach is in accordance with those previously reported in the literature for 3D printed tablets with channeled or gaplet designs based on poly-methacrylate (Eudragit E) or hydroxypropyl cellulose (HPC SSL) [37,40]. The study published by Sadia et al. investigated the impact of channel width, length, and alignment for a tablet design similar to ours on the drug release from the 3D printed structure and further disclosed that the characteristics of the channels have a significant influence on the dissolution pattern by affecting the flow of the medium through the gaps [37]. The referenced study concluded that a high number of shorter channels with a width of ≥0.6 mm were required to obtain an immediate release profile of the drug from the poly-methacrylate matrix. Based on these findings, we included short channels in our tablet design.

The variation of tablet geometry and structure is a simple strategy reported by several authors that allows the modulation of drug release from the 3D printed dosage forms [35,41,42]. Material selection remains, however, the fundamental consideration with respect to the desired release rate. A recent study by Ayyoubi et al. explored the effect of geometry on dissolution profiles when channeled mini-tablets based on either ethylcellulose or Kollidon VA 64 are prepared. An important outcome was that the increased surface area granted by the model significantly enhanced the dissolution only in case of the Kollidon VA 64 based tablets [43]. The greater surface area/volume ratio was not enough to substantially change the drug dissolution from the ethylcellulose structure. These results showed that the API release depended, firstly, on the composition of the tablet and only secondly on the geometry.

### 2.4. Solid State Characterization

**Figure 3:**
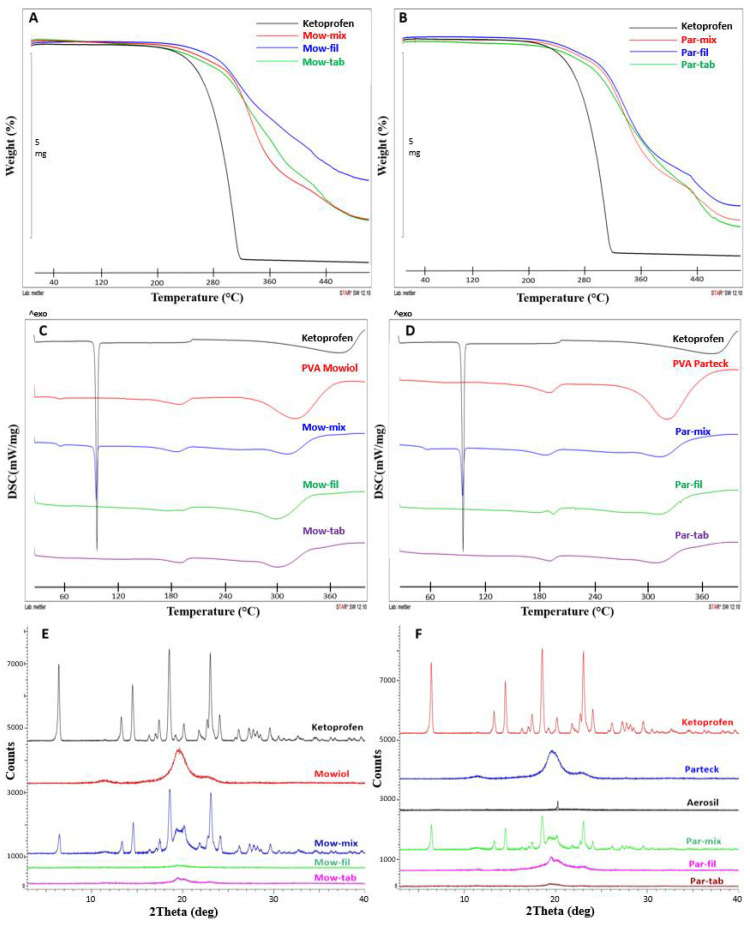
Physical characterization of raw materials, physical mixtures, filaments and 3DP tablets: (**A**,**B**) TGA profiles; (**C**,**D**) DSC thermograms; (**E**,**F**) X-Ray diffractograms.

Evaluating the degradation profile of the involved materials is indispensable for the orientation concerning selection of processing parameters in HME and FDM-3DP, as the thermal impact is a particularity of these technologies. Preserving the integrity of the API throughout the extrusion and printing phases is critical, considering its exposure to a series of instability factors including elevated temperatures, humidity, mechanical stress, and additives [44]. Thermogravimetric analysis (TGA) of the API, physical mixtures, filaments, and 3DP tablets (Figure 3A,B) disclosed that the applied processing conditions, i.e., 180 °C during HME and 185 °C during FDM-3DP, are not causing any decomposition of KET or excipients. According to the same measurements, unassociated API showed decomposition initiation at 200 °C, and the KET containing physical mixtures, filaments, and tablets followed the same pattern.

Differential scanning calorimetry (DSC) thermograms are presented in Figure 3C,D. Pure KET displays a melting endothermic peak at 94 °C, confirming the crystalline state of the API [45,46]. Thermograms of the two sorts of PVA displayed glass transition endotherms at 46 °C for MOW and 52 °C for PAR, while the T_m_ was depicted at 190 °C for each polymer. Analysis of the physical mixtures revealed the characteristic peaks of the components, confirming that the solid state of the materials was not modified by regular blending. The absence of the API’s melting endotherm in the filaments and 3DP tablets indicates the complete conversion of KET into an amorphous state, implying the formation of a one-phase system already in the HME step by processing the blends above the T_m_ of the drug which fuses with the carrier. The corresponding endotherm peaks representative for PVA were present in both filaments and tablets, regardless of the type of polymer used, the polymer maintaining its semi-crystalline state following thermal treatment.

X-ray diffraction (XRD) analysis was carried out in order to examine the solid-state conversions along the processing steps. Diffractograms of raw materials, physical mixtures, filaments, and 3DP tablets are presented in Figure 3E,F. The spectrum of pure KET illustrates numerous sharp diffraction peaks representative for its crystalline state [47,48], while the API containing filaments and 3DP tablets patterns are lacking these characteristic indicators, confirming the DSC findings regarding amorphization of KET during the first thermal treatment applied through HME phase. Considering that ASDs tend to reverse to their stable crystalline structure with the passage of time and the stability of the system constitutes a critical quality attribute [16,31], it is pertinent to note that XRD measurements were performed on filament and tablet samples prepared 6 months prior to analysis and kept in sealed plastic bags at room temperature. Despite the fact that fresh filaments and tablets were not analyzed by XRD in order to compare the results, since the stored samples revealed that KET remained in its unstable amorphous state and did not convert to the more stable crystalline form, it is appropriate to conclude that the physical stability of the system was maintained for the mentioned timeframe. These findings highlight the suitability of PVA as a polymer with the capacity to ensure solid state stability when an ASD is the sought outcome for drug solubility improvement and consecutive bioavailability enhancement.

**Figure 4:**
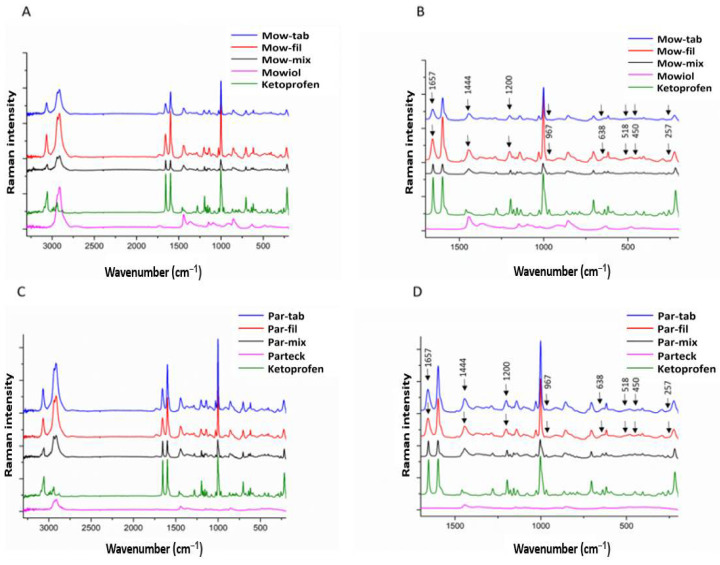
(**A**) Raman spectra of Mow, KET, and Mow-based formulations and (**B**) the magnification of fingerprint range spectrum; (**C**) Raman spectra of Par, KET, and Par-based formulations and (**D**) the magnification of fingerprint range spectrum.

Raman spectroscopy and mapping were completed for further physicochemical evaluation and assessment of the drug distribution in the filaments and the 3DP tablets. The spectra registered for both formulations were similar, as it is visible in Figure 4, differences occurring in the Raman intensity of the polymer, which increased in a proportionate manner with the particle size. The loss of the Raman bands in the spectra of both types of filaments and tablets at 257 cm^−1^, assigned to the τ(C^9^H_3_) and δ(C^9^H_3_-C^8^(C^10^=O)) API structure vibrations, and at 450, 518, and 638 cm^−1^ attributable to C^10^-O-H out-of-plane bending vibrations, is strongly confirming the participation of the CH_3_CHCOOH fragment of the API in the interactions established with the PVA molecules. Furthermore, the intensity decrease detected for the bands at 967 cm^−1^ (assigned to CH_3_ rocking and antisymmetric deformation) and at 1444 cm^−1^ (assigned to antisymmetric deformation) are in conformity with that premise, taking into consideration the immediacy of this methyl group to the interaction site. Clearly, a novel setting is generated for CH_3_ by the KET-PVA interaction. Additionally, the band at 1657 cm^−1^ resulted as a consequence of the stretching vibration of the inter-ring KET carbonyl ν(C^7^=O^13^), suffers a pronounced peak intensity reduction coupled to an increase in full width at half maximum in products. The almost generalized increase in the bandwidths in the difference spectrum indicates a significant loss of crystallinity of KET in the products, the spectral changes detected at 1657 cm^−1^ presumably resulting from an amorphization of the API in the presence of the matrix forming polymer, a phenomenon also revealed by the broadening of the bands due to the ring CH in-plane bending (e.g., 1200 cm^−1^). Comparing the Raman spectra of pure KET to those of filaments and 3DP tablets consisting of KET and PVA, the identified alterations appear to include two specific sections of the molecule, i.e., the methyl-carboxylic region and the inter-ring C^7^=O^13^ group, demonstrating the generation of hydrogen bonds between the API and the excipient [49]. KET altered the regular structure of PVA by disrupting the intra- and intermolecular interactions of the polymeric phase through the newly generated bonds, therefore, acting as a non-traditional plasticizer. Furthermore, the HME process enabled a dissolution-like phenomenon of the API in the polymeric carrier along with its amorphization. The hydrogen bonds established between PVA and KET maintained the physical stability of the system over time, by reducing the molecular freedom of movement of the API and increasing the resistance to recrystallization.

The above-described findings, namely API amorphization and dissolution in the polymeric carrier, are among the sought outcomes and advantages of the HME technology. Preparation of amorphous solid dispersions by HME is one of the strategies employed for the enhancement of the solubility and bioavailability of poorly soluble drugs such as KET, a BCS class II drug. The solubilization of the API in the polymer matrix under controlled processing conditions ensures mixing at a molecular level and the preparation of a thermodynamically stable single-phase system. In contrast, if the amorphization of the API is achieved but the amorphous drug is simply miscible with the polymer and no solubilization occurs, the system is less stable and exhibit a greater tendency to transform into a more stable state by crystallization [25].

**Figure 5:**
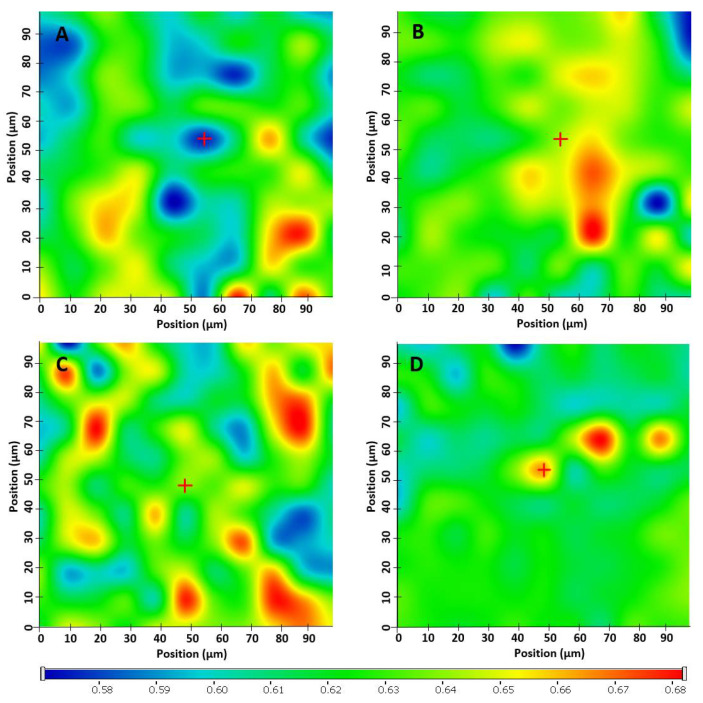
Raman mapping depictions of (**A**) Mow-fil, (**B**) Mow-tab, (**C**) Par-fil, (**D**) Par-tab.

Raman mapping was employed to examine the homogeneity of drug distribution in filaments and tablets. The characteristic bands obtained from Raman spectrum of pure KET as reference have been used to visualize the spatial distribution of the API in the samples. The relative intensity of the chemical maps was normalized to pure KET spectrum as a profiling reference. Profiling the spectrum of KET resulted in low changes in the relative intensity ratio (0.58–0.67) of the whole chemical maps (Figure 5). As the lowest intensity of KET detected was 0.58, it proves that KET was found in each measurement point. The intensity differences in the chemical maps are quite low, which proves the homogenous distribution of KET both in filaments and tablets.

### 2.5. Characterization of Filaments

Filament diameter uniformity is fundamental as variations affect the mechanical properties. Thin sections tend to fracture more rapidly than thicker ones [50], frequently leading to filament breakage inside the print head and disruption of the printing process. It also impacts the uniformity of mass of the 3D printed drug delivery systems, since in FDM the average diameter of the filament is applied for the determination of the feed rate in the G-code. Therefore, low diameter differences must be ensured to acquire 3D printing of reproducible drug products in agreement with the quality standards of the European Pharmacopoeia for single-dose preparations. The hot melt extruded filaments with 30% w/w KET were smooth and presented a mean diameter of 1.71 ± 0.08 mm in case of Mow-fil and 1.65 ± 0.04 mm in case of Par-fil. Utilization of conveyor belts has been reported in the literature as a useful tool for adjusting filament diameter. Thus, the filament diameter reported by Ilyes et al., was 1.65 ± 0.1 mm [51]. In comparison, although a conveyor belt was not utilized for the preparation of our filaments, the reduced values of the calculated standard deviations from the average width indicate appropriate uniformity.

Solubility is an intrinsic quality of the pharmaceutical active compound that has a significant implication in the absorption and hence the bioavailability of the drug. Formulation of ASDs is among the explored strategies to enhance oral drug delivery, leading to improved bioavailability of APIs with inadequate solubility. Since KET is a BCS class II drug with poor solubility and HME technology is employed for the preparation of ASDs, the impact of the filament preparation phase on the solubility of KET was assessed. Table 3 presents the results of saturation solubility studies for pure KET and KET-loaded filaments at pH 1.2, 6.8, and 7.4. As indicated, the pure API exhibited a solubility of 0.089 mg/mL at pH 1.2. However, as a result of processing the API containing polymeric mixtures by HME and subsequent amorphization of the drug, the solubility of KET at pH 1.2 was significantly increased to 2.723 mg/mL in Mow-fil and 0.825 mg/mL in Par-fil. Despite the fact that amorphization of KET was achieved and ASDs were obtained through the preparation of both Mow-fil and Par-fil, as it was revealed by the solid state evaluation of the samples by DSC and XRD, superior solubility enhancement was allegedly obtained for Mow-fil at pH 1.2 as a result of the prolonged intimate mixing of the components which ensured better solubilization of the drug in the polymeric matrix [52]. It has been also hypothesized that the non-covalent bonds, as for example hydrogen bonds, established within the API and the polymer during HME contribute to solubility enhancement [53]. Such interactions were evidenced by means of Raman spectroscopy (Figure 4) between KET and PVA in both types of filaments and tablets, but most likely the prolonged thermal processing in case of Mow-mix enabled superior solubility enhancement compared to Par-mix due to the extended length of the HME phase that allowed a higher occurrence of hydrogen bonds between the API and the polymer. In a recently published study, the matter of better amorphization of the API in a Kollidon VA64 based matrix than in one of Ethylcellulose was discussed [43]. In the referenced work, with respect to the Hansen solubility parameters, the authors concluded that the differences between the systems occurred due to the better miscibility and solubility of nifedipine in Kollidon VA64. By comparison, in our study, the two formulations were based on the same type of polymer (as both MOW and PAR are PVA 4-88) and we achieved amorphization of the API by HME in both formulations. However, as well as in the referenced study, differences in the solubilization of the API in the two PVA matrices presumably occurred. In our case, the solubility variations of KET in the polymeric matrices are explicable by the different extent of residence time of the mixtures during the extrusion. Prolonged thermal treatment and intimate mixing of Mow-mix led to a better solubilization of KET in the polymer, and consequently to a superior solubility enhancement in case of Mow-fil, compared to Par-fil. Ketoprofen extrudates presented slightly increased solubility compared to raw API at pH 6.8, but no substantial differences were found at pH 7.4.

The drug content analysis of the custom-made filaments was performed in phosphate buffer at pH 6.8 due to the pH-dependent solubility of KET that was highlighted by the previously presented solubility study carried out in different dissolution media (Table 3). The investigation established an efficient drug loading, as processing the physical mixtures through HME resulted in a 28 ± 2.4% API content for the Mow-fil and 28 ± 1.3% for the Par-fil, validating the homogenous distribution of KET evidenced by Raman mapping and the stability of the API during the HME step. As the API stability at the processing temperature was already established by means of thermal analysis, the increased variation in case of Mow-fil was expectable since previous works emphasized the importance of polymer particle size on the ability of the carrier to load the drug [26]. The finer PVA batch containing formulation exhibited slightly increased uniformity, finding which is in complete agreement with the literature.

### 2.6. Characterization of Printed Tablets

Pharmaceutical characterization of the 3DP dosage forms included examination of weight uniformity, resistance to crushing and disintegration (Table 4). Obtaining highly reproducible units in terms of weight and dimensions is of the utmost importance for pharmaceutical exploitation of the technology as uniformity of dosage units guarantees the delivery of the required dose of the API. It was previously highlighted in the literature that the quality of the FDM 3D printed items relies on a tight interplay between the characteristics of the involved materials and the operating parameters of the printer [54]. As presented in Table 4, the individual mass variations of the 3DP tablets were among reduced ranges and met the requirements of the European Pharmacopoeia for the “uniformity of mass of single-dose preparations” [55]. However, further increased accuracy could be achieved through optimization of the conformable machine settings. Resistance to crushing test was performed with the aim of determining the implications of the channeled model on the mechanical properties of the tablets, as the embedded gaps could stand for potential break points. However, the dosage forms showed elevated crushing strength, taking into consideration that by employing the maximum applicable force by the equipment, breaking did not occur. The prominent mechanical features were allegedly favored by performing the printing with 100% infill. Evaluation of disintegration revealed relevant variations between the two types of tablets, as it is indicated in Table 4. Presumably the slower disintegration behavior of Mow-tabs resulted from the prolonged residence time of the Mow-mix blend inside the barrel during the HME step due to the increased polymer particle size as compared to Par-mix, which likely contributed to a thermal crosslinking process among the monomeric units of the polymer, decreasing the hydrophilia of the matrix forming material [56,57]. According to the literature, the degree of crosslinking amplifies as the crosslinking temperature and the duration of thermal treatment increases [56]. Since the processing temperatures, i.e., 180 °C during HME and 185 °C during FDM-3DP, were identical for both formulations, along with the printing speed that was preset and kept constant, variations occurred solely in the residence time of the mixtures in the barrel during the filament preparation step. Consequently, following prolonged thermal treatment of Mow-mix, a darker colored filament was obtained, Mow-fil, respectively, and as a result, a darker colored Mow-tab was prepared by FDM-3DP (Figure 1). The more intense coloration indicates changes in the chemical structure of the PVA matrix, leading to a decreased solubility in water and slower disintegration [56,58].

**Figure 6:**
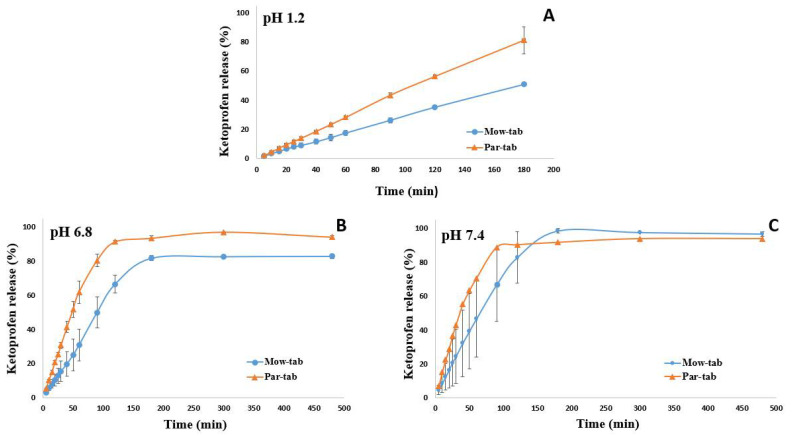
In vitro drug release patterns at (**A**) pH 1.2, (**B**) pH 6.8, and (**C**) pH 7.4 from 3D printed tablets.

In vitro release patterns from tablets containing ~245 mg of ketoprofen load and printed with 100% infill examined in three different pH media (pH 1.2, 6.8, and 7.4) are presented in Figure 6. Investigations were carried out in diverse buffers in order to assess the impact of pH on the dissolution performance of the tablets. The potential interference of the polymer with API absorption at the wavelength used for the spectrophotometric quantification of KET was excluded, as the UV-Vis spectrum of the polymer revealed no absorption at the employed wavelength.

It is observable in Figure 6 that the API dissolution begins promptly after the contact with the buffer solutions. In all three dissolution media a significant difference in the release behavior of the API from the two systems is perceptible, as the smaller particle size polymer containing formulation, Par-tab, respectively, displayed a faster drug release rate. This outcome is explicable by the reduced residence time of Par-mix in the extruder during the Par-fil filament preparation step. The small particle size of PAR ensured a more rapid melting of the mixture and reduced the required processing time at high temperatures. Thus, the diminished window of opportunity for thermal crosslinking to occur between the monomeric units of PVA in case of PAR based products better preserved the hydrophilia of the polymeric matrix and ensured a more rapid erosion that led to faster dissolution of KET from Par-tab compared to Mow-tab. These findings disclose novel considerations regarding material selection for a PVA-based system with regards to the desired characteristics of the final dosage form.

**Figure 7:**
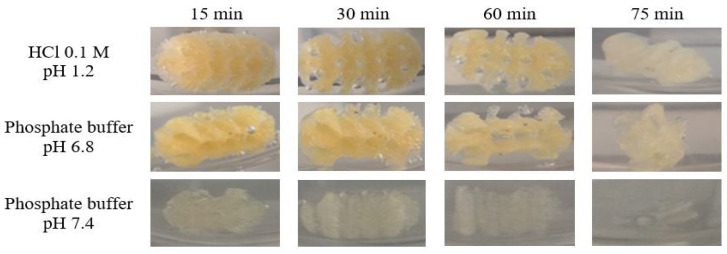
Depictions of KET-loaded FDM 3D printed channeled tablets throughout dissolution in HCl pH 1.2, phosphate buffer pH 6.8, phosphate buffer pH 7.4.

Previous investigation established that API release from the PVA matrix is controlled by erosion and diffusion processes [19,37]. As shown in Figure 6A, 35% of the drug was released from the Mow-tab into the hydrochloric acid media (pH 1.2) after 2 h, whereas 56% of the KET content was released by the Par-tab in the equivalent time frame. The dissolution profiles of the 3DP dosage forms in pH 6.8 phosphate buffer are presented in Figure 6B and it may be noted that the Mow-tab reached 67% drug release in 2 h, while in comparison the Par-tab reached over 90% release in 2 h. A similar trend was obtained in pH 7.4 dissolution media (Figure 6C) with the remark that in the instance of Mow-tab the plateau was attained in 3 h with complete drug release (100%), while the Par-tab achieved comprehensive dissolution after 90 min (~90%). The KET release profile kinetics were assessed by fitting the results to six mathematical models (zero-order, first-order, Higuchi, Baker and Lonsdale, Hixon and Crowell, and Peppas) and a good correlation and model prediction was found with a zero-order release profile in 0.1 M hydrochloric acid medium and Hixon and Crowell release profile in phosphate buffer media. The selected model drug is a weak acidic substance with pH-dependent solubility, and it is also observable that the release rate of the API is highly dependent on the pH of the media, as slower dissolution (Figure 6) and erosion of the tablets is noticeable in the acidic media (Figure 7).

Previous investigations demonstrated that the size of the 3D printed tablet has a pronounced effect on the dissolution process. According to the results published by Palekar et al., the PVA-based minicaplet with a length of 10 mm and 100% infill density reached 85% drug release in 100 min, while the design with a length of 5 mm and printed with 100% infill released 85% of the API in 18 min [21]. By comparison, our considerably larger tablets (Par-tab) released 90% of the drug in 90 min. Therefore, our results showed that by accurate design strategies, i.e., channeled tablets first reported by Sadia et al. for hydroxypropyl cellulose based 3D printed tablets [37], these drawbacks could be successfully surpassed, if faster release is desirable. Moreover, by adjusting the porosity of the 3D printed structure by lowering the infill percentage, the dissolution rate could be further accelerated [59], as previous studies that investigated the influence of the infill on the drug release of PVA based systems have shown [7,8].

## 3. Materials and Methods

### 3.1. Materials

The active pharmaceutical ingredient, ketoprofen, was kindly gifted by Cosma S.p.A. (Ciserano, BG, Italy). The matrix forming polymer, PVA with larger particle size Mowiol^®^ 4–88 (MOW, M_W_ ~ 31,000) was purchased from Sigma–Aldrich (St. Louis, MO, USA) and the finer sort Parteck^®^MXP (PAR, M_W_ ~ 31,000) was donated by EMD Millipore Sigma (Burlington, MA, USA). Fumed silica (Aerosil^®^) was acquired from Evonik Industries AG (Essen, Germany).

### 3.2. Material Characterization

#### 3.2.1. Particle Size Analysis

Preliminary particle size reduction in MOW was performed by grinding. The mean diameter of MOW and PAR particles was assessed by laser light diffraction, utilizing Analysette 22 MicroTec Plus equipment provided with a Dry Dispersion Unit component (Fritsch, Weimar, Germany).

#### 3.2.2. Flowability

The flow properties of MOW, PAR, and those of the physical mixtures with the qualitative and quantitative formulas presented in Table 1, Mow-mix and Par-mix, were evaluated by determination of Carr’s Index and Hausner ratio, derived from bulk and tapped density values of samples. Assessment of the bulk and tapped densities was carried out by using a powder density testing system (SVM-10 Erweka, Heusenstamm, Germany) [60]. Two repeated measurements were carried out for each batch of powders. Bulk density was calculated as the ratio of the weight of the powder sample to the volume read after pouring it into the cylinder, while the tapped density was determined by dividing the weight of the powder by tapped volume.

Carr’s index = (tapped density – bulk density)/tapped density × 100 [61]

Hausner ratio = tapped density/bulk density [61]

### 3.3. Preparation of Hot Melt Extruded KET-Loaded Filaments

Overall, two formulations were considered for processing by HME. The first one was based on MOW, the PVA sort with larger particle size, and the second was based on PAR, the finer batch of PVA. The constituents of each blend were pre-weighed according to the quantitative formulas presented in Table 1 and mixed in mortar with pestle, to obtain Mow-mix and Par-mix. Geometric dilution was employed in case of Par-mix, to ensure the uniform distribution of Aerosil. Each mixture was subsequently extruded to obtain the KET-loaded filaments, Mow-fil and Par-fil, respectively.

Extrusion of the API containing polymeric blends was carried out by a single-screw extruder (Noztek Pro, Noztek, UK) through a 1.75 mm die at a rotational speed of 65 rpm. Configuration of the extrusion system comprised a mixing zone and a heating compartment preceding the nozzle. Both formulations were processed at 180 °C and the feeding rate was set to 1.5–2 g/min. The optimization of the extrusion temperature was established by outcome aspect observing. The prepared filaments were stored at room temperature in sealed plastic bags to prevent moisture absorption.

### 3.4. Preparation of 3D Printed Tablets

The preparation of tablets by FDM-3DP relied on the KET-loaded filaments, Mow-fil, and Par-fil. Therefore, two types of tablets were obtained, Mow-tab and Par-tab, respectively. The printing process was performed using MakerBot Replicator 2X (MakerBot, Brooklyn, NY, USA). The channeled tablet model presented in Figure 1 was designed in Fusion 360 (Autodesk, San Rafael, CA, USA), saved as an .stl file that describes the surface geometry of the object and imported to MakerBot^®^Desktop Software. The dimensions of the dosage forms were set at X = 19.97 mm, Y = 9.99 mm, Z = 9.98 mm, the integrated channels had a diameter of 2.3 mm, and the infill percentage was kept at 100%. Printing was done at 185 °C through a 0.4 mm nozzle and other printer settings included standard resolution with disabled raft and support options, build platform temperature 80 °C, first layer print speed 30 mm/s, infill print speed 90 mm/s, and 0.2 mm layer height.

### 3.5. Solid State Evaluation

#### 3.5.1. Thermal Characterization

Thermo-analytical examinations of the samples (raw materials, physical mixtures, filaments, and 3DP tablets) included DSC and TGA. Assessments were conducted on filament and tablet samples produced 6 months prior to analysis and preserved in plastic sealed bags at room temperature. Filament and 3D printed tablet samples were prepared by cutting them into smaller fragments with a scalpel.

TGA investigations were carried out with a TGA SDTA 851^e^ thermobalance (Mettler Toledo GmbH, Greifensee, Switzerland) from 25 °C to 500 °C at a heating rate of 10 °C/min and using N_2_ as an inert purge gas (50 mL/min). Samples were weighed (4–6 mg) and placed in 70 µL open alumina crucibles. The TG curves were analyzed using Mettler Toledo STAR^e^ software with the purpose of assessing the degradation profile previous to and after the thermal treatment.

DSC analysis was performed using DSC 822 equipment (Mettler Toledo GmbH, Greifensee, Switzerland). Accurately weighed samples (2–4 mg) were placed in 40 µL standard aluminum pans with pierced lids. Measurements were conducted under dynamic nitrogen atmosphere (50 mL/min) and the thermal cycle involved examination in the temperature range 25 °C to 400 °C at a heating rate of 10 °C/min. Data were collected with Mettler Toledo STAR^e^ software and analyzed in order to assess physical state transformations during processing.

#### 3.5.2. X-ray Diffraction

XRD patterns of the drug, excipients, physical mixtures, cut fragments of hot melt extruded filaments and 3DP tablets were obtained using Bruker D8 Advance diffractometer (Bruker AXS GmbH, Karlsruhe, Germany) with Cu K λI radiation (λ = 1.5406 Å) and VÅNTEC-1 detector. The samples were analyzed at 40 kV and 40 mA. The angular range was 3° to 40° 2θ, at a step time of 0.1 s and a step size of 0.007°. The samples were positioned on quartz holders without any pretreatment by grinding of the filaments and tablets in order to prevent any disruption of the present crystal structures. The diffractograms were employed with the purpose of monitoring the API physical state transformations as a result of the thermal treatments involved in the processing steps.

#### 3.5.3. Raman Spectroscopy and Mapping

For the investigation of raw materials, filaments, and tablets fragments cut using a scalpel, a Thermo Fisher DXR Dispersive Raman instrument (Thermo Fisher Scientific Inc., Waltham, MA, USA) equipped with a CCD camera and a diode laser operating at a wavelength of 780 nm was used. Raman measurements were carried out with a laser power of 12 mW at 25 µm slit aperture size with an exposure time of 2 s and acquisition time of 6 s, for a total of 32 scans per spectrum in the spectral range of 3300 to 200 cm^−1^ with cosmic ray and fluorescence corrections. All spectra were baseline corrected and smoothed prior to evaluation. The distribution of KET was investigated by Raman chemical mapping of the formulations. The 100 µm * 100 µm sized surfaces were analyzed with step size of 10 µm with an exposure time of 2 s and acquisition time of 4 s, for a total of 4 scans per spectrum. The Raman spectra were normalized in order to eliminate the intensity deviation between the measured areas.

### 3.6. Morphological Investigation

Surface characterization of hot melt extruded filaments and 3D printed tablets was performed by scanning electron microscopy (SEM). The samples were mounted on carbon double adhesive tape, attached to brass stubs and coated with a layer of gold. Image acquisition was conducted on a Hitachi SU8230 High Resolution Scanning Electron Microscope equipped with a cold field emission gun. The microscope was operated at 30 kV in both low and high magnification mode. Approximately 90% of each the sample was scanned to give a realistic overview of its morphology, and only a few representative areas were captured.

### 3.7. Characterization of HME Filaments

#### 3.7.1. Assessment of Filament Diameter

First of all, the aspect of the filaments was visually examined. Their diameters were measured shortly after extrusion as a quality control assessment using a digital caliper in six locations, every 25 cm (over a total length of 1.5 m).

#### 3.7.2. Solubility of KET-Loaded Filaments in Different Dissolution Media

Solubility studies of KET as a reference and KET-loaded filaments were carried out by adding a surplus quantity of pure drug and drug loaded filaments in 4 mL of 0.1 M hydrochloric acid medium at pH 1.2, phosphate buffer at pH 6.8 and phosphate buffer at pH 7.4. Two fragments from each type of filament and two reference KET samples were evaluated. The samples were kept in a thermostatic bath (Raypa Trade, Barcelona, Spain) at 37 ± 0.5 °C for 48 h and intermittently agitated and ultrasonicated (Transsonic T700, Elma, Germany). The samples were subsequently centrifuged at 10,000 rpm for 10 min. The supernatant was filtered via 0.22 µm filters (Phenomenex, Torrance, CA, USA), properly diluted and analyzed for drug content by spectrophotometric method (Specord^®^200 Plus, AnalytikJena, Jena, Germany) at λ = 255 nm.

#### 3.7.3. Assessment of Filament Drug Loading

A stock solution (3 mg/mL) of KET was prepared and further diluted with phosphate buffer at pH 6.8 in order to create seven solutions that were employed in the calibration curve construction. Phosphate buffer pH 6.8 was chosen as the diluent considering that KET solubility is pH dependent and increases promptly above pH 6 [29].

With the purpose of assessing drug content in the filaments, two fragments of approximatively 200 mg from each type of filament were placed in 500 mL phosphate buffer pH 6.8 and subjected to stirring and sonication until complete dissolution was achieved. Following proper dilution with the same buffer, samples were filtered through 10 µm cannula filters (Dissolution Accessories, München, Germany) and the filtrate was used to quantify KET by spectrophotometric determination at λ = 255 nm.

### 3.8. Pharmaceutical Characterization of 3D Printed Tablets

#### 3.8.1. Weight Uniformity

With the aim of assessing weight uniformity, six tablets were arbitrarily chosen for each formulation and weighed by operating high-precision digital balance (Ohaus^®^ Analytical Plus balance, resolution 0.01 mg). The average mass was calculated, and the standard deviation was determined for both preparations.

#### 3.8.2. Resistance to Crushing

The influence of the channeled design on the mechanical properties of the FDM 3D printed tablets was assessed by employing a crushing strength tester (PTB 111 E, Pharma Test, Hainburg, Germany). Then, five tablets were randomly selected from each formulation. Measurements were carried out by positioning the long axis of the dosage form on the trajectory of the applied force.

#### 3.8.3. Disintegration

The disintegration behavior of the channeled tablets was investigated by employing a QC-21 Hanson disintegration examining equipment (Hanson Research, Chatsworth, CA, USA). Then, two tablets from each formulation were arbitrarily chosen and the units were individually positioned into a compartment of the basket rack assembly, which was submerged into a beaker that contained distilled water maintained at 37 °C. The conclusive moment of the disintegration process for each dosage form was considered the point when no visible tablet fragments were detected in the mesh.

#### 3.8.4. Dissolution

In vitro drug release studies of the 3D printed tablets with a drug content of ~245 mg were carried out on PT-DT70 apparatus (Pharma Test Apparatebau AG, Hainburg, Germany) using USP Type II dissolution model (paddle). Then, two tablets from each formulation were randomly selected to be examined in each dissolution medium. Briefly, the dosage forms were placed in the vessel and stirred (50 rpm) in three different dissolution media (900 mL), at 37 °C, as follows: 0.1 M hydrochloric acid buffer (pH 1.2) for 3 h, phosphate buffer at pH 6.8 for 8 h, and phosphate buffer at pH 7.4 for 8 h. Samples (5 mL) were withdrawn from the dissolution medium at preset time points (5 min, 10 min, 15 min, 20 min, 25 min, 30 min, 40 min, 50 min, 60 min, 90 min, 120 min, 180 min, 300 min, 480 min) and replaced with fresh medium, then were filtered via 10 µm cannula filters and assayed using UV-VIS spectrophotometer (Specord^®^200 Plus, AnalytikJena, Jena, Germany) at 255 nm wavelength.

## 4. Conclusions

HME-FDM-3DP is a versatile tool for pharmaceutical manufacturing purposes, providing the opportunity to fabricate unique drug delivery systems through complexity and allowing achievement of various drug release profiles by careful selection of the materials, the 3D design and the processing conditions. In order to attain the desired outcome, design optimization and good management of the process parameters is required, based on consolidated product and method understanding.

The current study disclosed a new perspective regarding material considerations when a PVA-based FDM 3D printed system is the sought outcome. A first finding was that the enhancement of filament printability could be granted by a promptly melting API at the employed temperature, by fulfilling plasticizing functions. Avoiding the utilization of supplementary processing aids is of utmost importance if an ASD is the target formulation, as plasticizers may negatively influence the stability of the system. Secondly, the polymer particle size was highlighted as a significant factor influencing the residence time during the HME step, but also with further implications on drug release performance of the dosage forms. Therefore, if the final goal in case of a PVA containing formulation is the rapid release of the API, a reduced residence time during HME should be ensured by selecting polymer batches with small particle sizes and assuring adequate flow properties. Furthermore, the benefits of a channeled design for enhancing drug release from PVA-based tablets was emphasized.

The suitability of PVA as a matrix forming material for 3D printed tablets was once again demonstrated, this time on the grounds of its ability to maintain the stability of the API following amorphization, for at least 6 months at room temperature. Thus, our findings provide a novel insight into formulation strategies of FDM-3DP manufactured drug delivery systems.

## Figures and Tables

**Table 1:** Physical mixture compositions.

Blend Name	Ketoprofen (%)	Mowiol 4–88 (%)	Parteck MXP (%)	Aerosil (%)
Mow-mix	30	70	–	–
Par-mix	30	–	68.5	1.5

**Table 2:** Evaluation of flow properties by means of Hausner ratio and Carr’s index.

Evaluated Properties	Material		Formulation	
	MOW	PAR	Mow-Mix	Par-Mix
Bulk density (g/cm^3^)	0.70 ± 0.01	0.56 ± 0.005	0.57 ± 0.02	0.38 ± 0.01
Tapped density (g/cm3)	0.76 ± 0.01	0.75 ± 0.0	0.7 ± 0.01	0.5 ± 0.0
Hausner ratio	1.09 ± 0.004	1.25 ± 0.01	1.23 ± 0.03	1.33 ± 0.04
Carr’s index %	8 ± 0.4	20 ± 0.7	19 ± 1.7	25 ± 2
Flowability	excellent	fair	fair	passable

**Table 3:** Solubility of KET from API powder and API loaded filaments at pH 1.2, 6.8, and 7.4, expressed as mean saturation concentration (mg/mL) ± SD, n = 2.

pH	KET Solubility from Powder (mg/mL)	KET Solubility from Mow-Fil (mg/mL)	KET Solubility from Par-Fil (mg/mL)
1.2	0.09 ± 0.01	2.7 ± 0.1	0.8 ± 0.05
6.8	4.9 ± 0.02	6.7 ± 0.15	6.3 ± 0.3
7.4	7.8 ± 0.05	6.9 ± 0.15	7.6 ± 0.9

**Table 4:** Summary of pharmaceutical evaluation of 3DP tablets.

Tablet	Average Mass ± SD (mg), n = 6	Deviation from the Average Mass (%)	Breaking Force (N)	Disintegration Time ± SD (min), n = 2
Mow-tab	827.3 ± 9.1	−1.97 +0.93	>300	115 ± 6
Par-tab	805.2 ± 16.2	−1.76 +2.71	>300	83 ± 5

## Data Availability

The data presented in this study are available in this article.
